# Destabilizing single chain major histocompatibility complex class I protein for repurposed enterokinase proteolysis

**DOI:** 10.1038/s41598-020-71785-2

**Published:** 2020-09-10

**Authors:** Jackwee Lim

**Affiliations:** Singapore Immunology Network, A*STAR, 8a Biomedical Grove, Singapore, 138648 Singapore

**Keywords:** Biochemistry, Biotechnology, Immunology

## Abstract

The lack of a high throughput assay for screening stabilizing peptides prior to building a library of peptide-major histocompatibility complex class I (pMHC-I) molecules has motivated the continual use of in silico tools without biophysical characterization. Here, based on de novo protein fragmentation, the EASY MHC-I (EZ MHC-I) assay favors peptide antigen screening to an unheralded hands-on time of seconds per peptide due to the empty single chain MHC-I protein instability. Unlike tedious traditional labeling- and antibody-based MHC-I assays, repurposed enterokinase directly fragments the unstable single MHC-I chain protein unless rescued by a stabilizing peptide under luminal condition. Herein, the principle behind EZ MHC-I assay not only characterizes the overlooked stability as a known better indicator of immunogenicity than classical affinity but also the novel use of enterokinase from the duodenum to target destabilized MHC-I protein not bearing the standard Asp-Asp-Asp-Asp-Lys motif, which may protend to other protein instability-based assays.

## Introduction

**Figure 1:**
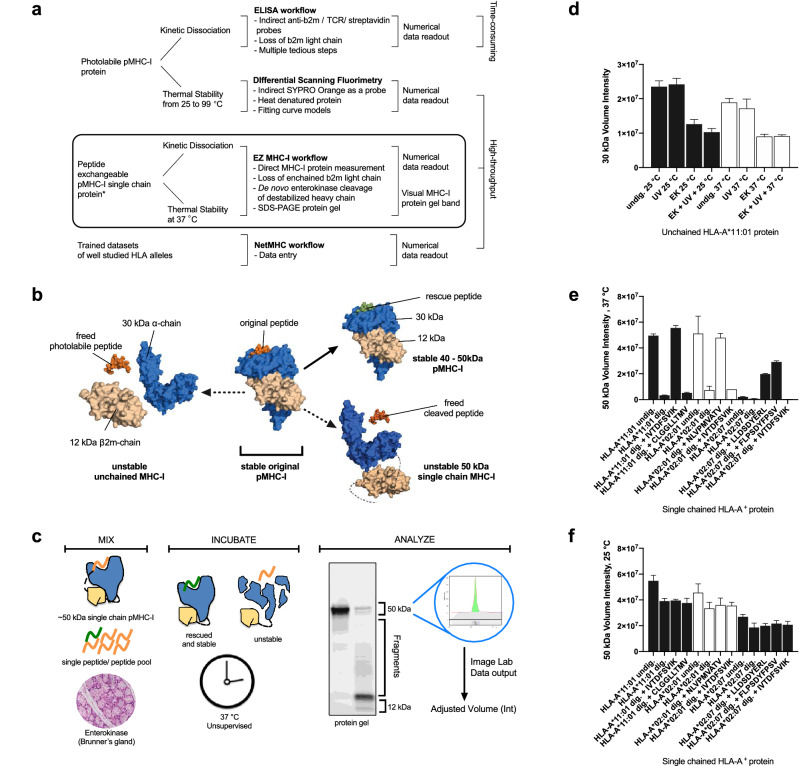
The destabilized single chain MHC-I protein is directly digested by enterokinase to ease stabilizing peptide screening. (**a**) Comparisons among pMHC-I stability-based assays such as traditional ELISA workflow, differential scanning fluorimetry (NeoScreen Technology, Immunitrack), NetMHC(stab-1.0) algorithm and EZ-MHC-I assay. (**b**) Empty MHC-I protein destabilization. In the absence of the original peptide either photolabile or cleavable, both forms of empty unchained MHC-I protein will dissociate unless stabilized by a rescue peptide. In the empty single chain MHC-I protein, the leaving β2m further destabilizes the enchained α chain. (**c**) EZ MHC-I workflow. Mix the single chain pMHC-I protein, the peptide of interest (preferably single peptide) and intestinal enterokinase in the same tube, incubate at 37 °C and detect fragments in a protein gel. The enterokinase preferably cleaves the unstable single chain MHC-I protein and will result in the loss of a 50 kDa protein band, which is quantified using the Image Lab software version 6.1 (Bio-Rad). (**d**) A plot of raw data of the stability of the unchained MHC-I protein bearing a photolabile peptide under varying conditions based on the volume intensity. About ~ 50% of 30 kDa α chain still remains as a protein band under different UV, thermal and enterokinase treatment. (**e**,**f**) A plot of raw data of the stability of the single chain MHC-I protein bearing a cleavable peptide in the presence of rescue peptides at pH 6.2 and 37 °C or 25 °C. HLA-A*11:01, HLA-A*02:01 and HLA-A*02:07 were digested without or with enterokinase. Significant improvement of up to 95% enchained MHC-I protein fragmentation at 37 °C instead of at 25 °C and is better than unchained photolabile MHC-I protein of only ~ 50% fragmentation. The positive peptide controls are IVTDFSVIK (HLA-A*11:01), NLVPMVATV (HLA-A*02:01) and LLDSDYERL or FLPSDYFFSV (HLA-A*02:07) alongside negative controls CLGGLLTMV for HLA-A*11:01 and IVTDFSVIK for both HLA-A*02:01 and HLA-A*02:07.

Human leukocyte antigen (HLA) A, B and C are genes of the highly polymorphic MHC-I proteins found on the surface of almost all cells. As part of the adaptive immunity, every antigen-derived peptide loaded on each MHC-I variant on the non-professional antigen presenting cells is unique, to stage a cytotoxic CD8^+^ T cell immune response against foreign viruses, intracellular bacteria and self-tumors or even develop peripheral immune tolerance^1–3^. However, the peptide repertoire involves several classical antigen presentation and cross-presentation pathways^4^. Besides peptides from intracellular antigen, extracellular antigens when internalized can involve either vacuolar or endo/lysosomal proteasomal processing, which further increases the peptide possibilities on MHC-I proteins. Despite recent advances in the generation of large pMHC multimer libraries, selecting the peptides remains a significant challenge. Thus time-saving bioinformatics tools such as NetMHCpan-4.0 algorithm in constant development are still widely used to predict peptide candidates based on affinity for these pMHC multimer technologies^5,6^. However, retaining the peptide bound to the MHC-I protein requires stable pMHC-I molecules while waiting for specific but rare cytotoxic T lymphocytes. Indeed it has been shown that stability is a better indicator of immunogenicity and immunodominance^7^. As such, high-throughput NetMHCstab-1.0 prediction algorithm and NeoScreen platform have been introduced but these methods measure pMHC stability indirectly, using trained databases or molecular probes (Fig. 1a). Here, to directly characterize the pMHC-I protein, the EZ MHC-I technology is introduced using a repurposed enterokinase from the duodenum to fragment the α heavy chain of unstable single chain pMHC-I proteins but not the same α heavy chain of stable single chain pMHC-I protein. The actual MHC-I protein fragments are directly visualized on standard SDS-PAGE protein gel, with hassle-free workup steps, greater confidence and could potentially discover more stabilizing peptides as effective vaccine antigens that still remain a global challenge.

## Results

Following previous work, despite polymorphism among MHC-I protein family members, a common mechanism involves the highly conserved beta-2-microglobulin (β2m) protein to dissociate from the empty MHC-I protein complex and subsequent lysosomal proteolysis^8–10^. Thus in a hypothesis for a single chain MHC-I protein architecture, the leaving β2m protein will pull and expose residues to further destabilize the enchained α chain (Fig. 1b)^11^. Additionally, enterokinase protease was found to cleave the heavy chain of the unstable single chain MHC-I protein and not the stable one at 37 °C. Also, the newfound enterokinase preference for a more accessible unstable (conformational) state may also include unbound peptide at 37 °C. Therefore, the de novo protein fragmentation driven by enterokinase becomes the core of EZ MHC-I assay and is completed in three simple steps; mix, incubate and analyze (Fig. 1c). The resulting protein fragments are both visual and likely observed across the highly polymorphic MHC-I family, as represented by the frequent HLA-A^+^ alleles, A*11:01, A*02:01 and A*02:07 single chain proteins but not unchained one (Fig. 1d,e). In this work, the well-characterized HLA-A*11:01 will be used to represent the polymorphic MHC-I protein family for comparing the EZ MHC-I assay against existing methods and later the development of understudied alleles such as HLA-A*02:07, which cannot be accurately predicted.

As a proof-of-principle study, the likelihood of enterokinase induced protein fragments is first explored with two forms of conditional HLA-A*11:01 pMHC-I protein, unchained and single chain. The unchained pMHC-I protein consists of three molecules, an original peptide, the highly conserved β2m and the polymorphic α polypeptide chains whereas the same three molecules when separated by a spacer makes the single chain protein^11,12^. For the unchained HLA-A*11:01 protein bearing a photo-labile peptide, only 30 to 50% of the whole α chain fragmented under different temperature, UV and enterokinase conditions (Fig. 1d; Supplementary Fig. S1)^12^. This suggests a number of unchained MHC-I proteins still resistant to enterokinase even at 37 °C. On the contrary, the single chain pMHC-I protein bearing an enterokinase cleavable peptide showed an improved 70–95% of the protein fragmented at 37 °C unless rescued by a suitable peptide. These protein fragments originate from the α polypeptide chains, which do not bear the DDDD|K motif in the HLA-A*02:01, HLA-A*02:07 and HLA-A*11:01 proteins (Fig. 1e; Supplementary Fig. S1). Also, the absence of such protein fragments at 25 °C suggests that the enterokinase specificity was repurposed only at higher temperature (Fig. 1e,f). Thus secreted enzyme-exchangeable single chain proteins bearing cleavable bulging peptides from the insect cells were purified and the EZ MHC-I assay performed at 37 °C.

**Figure 2:**
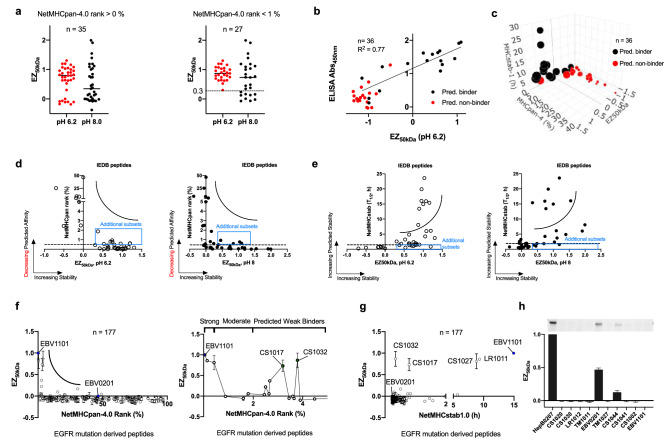
EZ MHC-I assay reveals additional IEDB peptides, which are not predicted strong candidates. (**a**) The pH effect on EZ MHC-I assay using 35 peptides retrieved from the IEDB resource and HLA-A*11:01 single chain protein. The NetMHCpan-4.0 cutoff is set as greater than 0% for all binders or less than 1% for potential binders (see Table 1). At pH 6.2, a tighter group of peptides is observed than at pH 8.0. (**b**) Comparison of traditional ELISA and EZ MHC-I assay with a collection of both IEDB peptide epitopes and peptides predicted using NetMHCpan-4 algorithm. Predicted non-binders tend not to be stabilizing whereas some predicted binders are found not stabilizing in both ELISA and EZ MHC-I assays (see Table 2). (**c**) A 4-dimensional comparison showing good agreement among the four methods. The bubble size is based on the ELISA assay whereby bigger size correlates to higher stability. The more stable and better binding peptides are clustered away from the less stable and poor binding peptides. Moderately predicted peptides may be confirmed using stability-based assays (see Table 2). (**d**) Additional peptides from IEDB resource are found stabilizing despite their higher NetMHCpan-4.0 ranks above 0.5%. (**e**) Additional peptides from IEDB resource are observed stabilizing despite falling below the recommended NetMHCstab-1.0 cutoff of 2 h. (**f**) A comprehensive EGFR peptide library (n = 177, duplicated) from key driver mutations were compared using EZ_50kDa_, NetMHCpan and NetMHCstab tools using HLA-A*11:01 single chain protein. The positive EBV1101 and negative EBV0201 control peptides are labeled and colored blue. Two more EGFR mutation-derived peptides (CS1012 and CS1017) are found strongly stabilizing despite their predicted weak affinities using NetMHCpan-4.0 algorithm for HLA-A*11:01. (**g**) Two stabilizing peptides (CS1012 and CS1017) were also not identified using NetMHCstab-1.0 algorithm based on its recommended cutoff of 2 h. (**h**) Analysis for understudied HLA-A*02:07 protein with peptides from the EGFR peptide library. The top eleven peptides with ascending NetMHCpan ranks are in the order of HepB0207 (FLPSDYFPSV, 0.028), CS1020 (TQLMPFGSLL, 0.086), CS1030 (ITQLMPFGSLL, 1.078), TM1011 (STVQLIMQL, 1.282), LR1012 (ITDFGRAKL, 1.534), EBV0201 (CLGGLLTMV, 1.596), TM1027 (MQLMPFGCLL, 2.194), CS1044 (QLMPFGSLLDYV, 2.238), CS1041 (LITQLMPFGSLL, 3.090), CS1002 (QLMPFGSL, 3.149) and EBV1101 (IVTDFSVIK, 11.64). For understudied HLA-A*02:07, there is a lack of correlation between NetMHCpan algorithm and measured EZ_50kDa_ stability (n = 11, duplicated). HepB0207 (FLPSDYFPSV) is a known positive control. The cutoff for EZ_50kDa_ at 0.3 is based on IEDB peptides with NetMHCpan-4.0 rank less than 1% and NetMHCstab-1.0 rank greater than 2 h (see Supplementary Fig. S3). Refer to Supplementary Fig. S2 for full gels of insert in (**h)**.

Next, for establishing that immunogenic peptides are also stabilizing or even resistant to enterokinase cleavage when bound to MHC-I protein, 35 known immunogenic peptides were retrieved from the Immune Epitope Database (IEDB, https://www.iedb.org/) (Table 1). In this work, a comparison was made for a trinity of methods, the EZ MHC-I assay using the HLA-A*11:01 single chain protein, the stability-based NetMHCstab-1.0 algorithm and the widely used affinity-based NetMHCpan-4.0 algorithm (Table 1). Unlike NetMHCpan, NetMHCstab algorithm could better predict stable T-cell epitopes instead of affinity matched-non epitopes but has not been updated due to the lack of experimental stability data^13^. Here, an inverse relationship is shown whereby increasing stabilities correspond to decreasing NetMHCpan rankings, which are higher peptide affinities. The EZ MHC-I assay classifies poor HLA-A*11:01 stability based on a different HLA-A*02:01 peptide score of zero (CLGGLLTMV) from the Epstein-Barr virus (EBV) proteome. Excellent HLA-A*11:01 peptide stability will be similar to a known antigenic HLA-A*11:01 peptide (IVTDFSVIK from EBV) score of one. To better understand this relationship using EZ MHC-I assay, 35 peptides from the IEDB resource were reduced to 27 with a smaller NetMHCpan cutoff of less than 1% to exclude poor affinity binders and is better performed at pH 6.2 (Fig. 2a). These 27 peptide epitopes generally have good EZ_50kDa_ stability scores between 0.3 and 1.0. And unlike the excluded eight peptides, which also had low stability NetMHCstab scores of less than 2 h, which would classify them as non-HLA-A*11:01 peptides (Fig. 2a; Table 1). Based on the IEDB resource, the reported MHC alleles for the eight unstable peptides are GPISGHVLK (HLA-A11, HLA-A3), TMVMELIRMIK (HLA-A*11:01), LVSFLLLAGR (HLA-A*11:01, HLA*03:01, HLA-A*31:01, HLA-A*33:01, HLA-A*68:01, HLA-A*30:01), LALEVARQKR (HLA-A*11:01), LVTFLLLCGR (HLA-A*11:01), LYASPQLEGF (HLA-A*11:01, HLA-A*24:02), AYQKRMGVQM (HLA-A*11:01, HLA-A*24:02) and negative control CLGGLLTMV (HLA-A*02:01) with variable response frequencies, including negative outcome reported in IEDB resource and thus possible lack of immunodominance. Next, EZ MHC-I assay was compared with traditional enzyme-linked immunosorbent assay (ELISA) using unchained protein. For ELISA, the single chain protein cannot be used as it mandates the separation of unchained α protein while the β2m protein remains attached to the ELISA plate. Unlike enterokinase-based EZ–MHC-I assay, the unchained protein bears a photo-labile peptide whose complete cleavage depends on efficient UV irradiation while assuming minimal protein photo-damage. Despite these technical differences, there is still a good agreement between ELISA and EZ-MHC-I assays whereby the NetMHCpan predicted good affinity binders are stabilizing but not the non-affinity binders (Fig. 2b). Moreover, this pattern of good affinity binders that are also stabilizing is conserved across all four methods (Fig. 2c; Table 2). However, deeper analysis with the EZ MHC-I assay also highlighted an additional number of immunogenic peptides, which were missed in NetMHCpan and NetMHCstab algorithms. These moderate peptides would be excluded as both predictive algorithms heavily rely on experimental data, which are often biased towards strong HLA affinity but may be experimentally identified using stability-based assay (Fig. 2d,e). Moreover, more suboptimal peptides are found stabilizing at pH 6.2 than pH 8 in EZ MHC-I assay (Fig. 2d,e). Hence, EZ MHC-I assay can detect stabilizing peptides and despite the possibility that enterokinase may target non-DDDD|K peptides, it did not lessen the number of identified immunogenic peptides but perhaps more than predictive algorithms at pH 6.2. This is probably due to the conformational protection when these labile peptides become inaccessible to enzymatic cleavage when bound to the MHC-I protein.

**Table 1:** HLA-A*11:01 characterization of peptide epitopes at two different pHs retrieved from the Immune Epitope Database (IEDB) with EZ MHC-I assay, NetMHCpan-4.0 and NetMHCstab-1.0 algorithms.

Index	SEQUENCE	IEDB	Origin	NetMHCpan-4 Rank (%)	NetMHCstab-1 Score (T_1/2_, h)	EZ_50kD_ (pH 6.2)	EZ50_kD_ (pH 8)
1	ATIGTAMYK	5,002	EBV	0.0026	23.5793	1.031625511	1.190285064
2	ATYGWNLVK	5,223	DENV1	0.0049	8.0727	0.870605547	1.994035494
3	STYGWNIVK	180,757	DENV3	0.005	4.8528	0.630630693	1.394508024
4	TTYLGPLSCK	94,098	LCM	0.0399	6.0105	1.083751608	1.911496583
5	IVTDFSVIK (EBV1101)	29,466	EBV	0.0423	14.9481	1	1
6	ATVQGQNLK	5,200	CMV	0.044	13.4333	0.896765373	0.766999991
7	AVQTKPGLFK	150,153	DENV2	0.0496	16.0378	0.955015006	1.546255991
8	KTFVDLMRR	124,391	DENV2	0.086	1.4929	0.82408852	0.165047447
9	KSGAIKVLK	180,577	DENV3	0.133	1.8625	0.818964843	0.48653242
10	AINSEMFLR	175,443	IGRP*	0.134	1.5431	0.901224151	1.189880277
11	RVIDPRRCLK	150,556	DENV1, 3, 4	0.15	15.9042	1.07454914	0.733917026
12	TSGSPIIDK	150,660	DENV2	0.169	1.968	0.795327602	0.159605484
13	RQLANAIFK	55,437	DENV3	0.17	3.7199	1.303197077	1.118237914
14	MSTYGWNIVK	180,674	DENV3	0.181	1.8548	0.642319424	0.125122315
15	MATYGWNLVK	180,645	DENV1, 4	0.208	1.3226	0.638430333	0.007553278
16	RVIDPRRCMK	56,310	DENV2	0.21	15.4	1.16173446	0.346334735
17	SVQPTFSVQR	144,488	Influenza A	0.31	4.2372	1.051411878	0.7376486
18	VTRGAVLMHK	150,715	DENV2	0.393	1.9538	0.622778602	− 0.068851052
19	YVSAIAQTEK	150,751	DENV2	0.437	2.5672	0.303719293	− 0.063560293
20	TTKRDLGMSK	180,788	DENV3	0.477	1.4143	0.79595639	0.379550736
21	MANEMGFLEK	150,431	DENV2	0.5519	3.2158	0.526954362	− 0.019570479
22	SSCSSCPLSK	60,930	EBV	0.585	19.8885	0.957571019	0.840850452
23	MVSRLLLNR	180,678	DENV3	0.592	0.8642	1.195884297	0.23955557
24	MSYTMCSGK	180,676	DENV4	0.6211	6.1212	1.19750423	1.094062057
25	GAMLFLISGK	180,469	DENV3	0.76	2.3863	0.746753143	1.036357414
26	FTNDSIISH	92,757	LCM	0.798	1.2262	0.696137865	0.133868081
27	TLALEVAQQK	175,503	Insulin-2	0.93	1.021	0.739914818	0.451747794
**Peptides with NetMHCpan-4 Rank > 1.0%**							
28	*GPISGHVLK	21,665	CMV	2.5	0.6046	− 0.065088414	− 0.05275318
29	*TMVMELIRMIK	566,925	Influenza A	4.98	0.86	− 0.10894061	− 0.004370881
30	*LVSFLLLAGR	93,350	LCM	5.86	0.6594	− 0.184620433	0.072058808
31	*LALEVARQKR	175,477	Insulin-1	8.86	0.6903	− 0.10544474	− 0.012738075
32	*LVTFLLLCGR	119,268	Lassa virus	9.38	0.7715	− 0.173186487	− 0.376760316
33	*LYASPQLEGF	176,343	Influenza A	25.231	0.2322	− 0.699688536	− 0.061802853
34	*AYQKRMGVQM	175,777	Influenza A	27.1	0.25	− 0.102005257	− 0.081022458
35	CLGGLLTMV (EBV0201)	6,568	EBV	46.6	0.2538	0	0

For EZ MHC-I assay, the EZ_50kDa_ score for each peptide is calculated using positive Epstein-Barr virus peptide control (EBV1101) and negative Epstein-Barr virus peptide control (EBV0201); ranging from above 1 (more stable than control), 1 (equally stable) to below 0 (very unstable), determined with Eq. (4). The most optimal peptides are scored closer to ~ 0 in NetMHCpan rank, greater than 2 h for NetMHCstab-1.0 half-life and greater than 0.3 for EZ_50kDa_. The seven peptides with EZ_50kDa_ below EBV0201 (negative control) as shown in Fig. 2a, are marked with * in Table 1.

*EBV* Epstein–Barr virus, *DENV* Dengue virus, *LCM* lymphocytic choriomeningitis virus**,** *CMV* cytomegalovirus**,** *IGRP* Islet-specific glucose-6-phosphatase catalytic subunit-related protein.

**Table 2:** HLA-A*11:01 characterization of peptide epitopes retrieved from the Immune Epitope Database (IEDB) and putative peptides with ELISA, EZ MHC-I assay, NetMHCpan-4.0 and NetMHCstab-1.0 algorithms.

Index	SEQUENCE	IEDB	Origin	NetMHCpan-4 Rank (%)	NetMHCstab-1 Score (T_1/2_, h)	EZ_50kD_ (pH 6.2)	ELISA_450nm_
1	RSFKDLLKK	140,739	Toxoplasma gondii	0.029	7.95	0.609199079	1.682000041
2	TVMDIISRR	150,674	DENV2	0.047	4.48	1.086457267	1.944100082
3	NTLEQTVKK	922,935	Papillovirus	0.052	9.78	0.564723004	1.631200015
4	GTSGSPIIDK	22,818	DENV1	0.08	6.45	0.633428224	1.385050059
5	ATVLMGLGK	150,147	DENV2	0.109	5.66	1.038798618	1.910549939
6	AVFDRKSDAK	5,316	EBV	0.125	26.36	0.624952021	1.078250051
7	HTLWKAGILYK	24,943	HBV	0.126	9.1	0.442617707	1.58130002
8	SSCSSCPLSK	60,930	EBV	0.191	19.89	0.664854145	1.272400141
9	STIPETTVVR	N.D	HBV	0.234	2.56	0.322579265	1.391299963
10	STLPETTVIR	N.D	HBV	0.274	3.33	− 0.113447642	1.302249968
11	RTLSPRRRR	N.D	HBV	0.422	2.12	− 1.143584408	0.865800023
12	*RTQSPRRRR	922,980	HBV	0.723	1.41	− 0.686832534	1.20449996
13	*SQSPRRRSK	N.D	Leptomonas seymouri	0.832	0.72	− 0.986875791	0.256799996
14	SAICSVVRR	56,814	HBV	0.917	1.77	0.238176504	1.618999958
**Peptides with NetMHCpan-4 Rank > 1.0%**							
15	*KFLPDLYDYK	144,377	Influenza A	1.325	0.83	− 0.748050496	0.480850041
16	^#^LVVDFSQFSR	40,624	HBV	1.782	3.37	0.946052213	1.45175004
17	*RTLSPRRR	N.D	Streptomyces sp	3.529	2.78	− 0.986037136	0.120900035
18	*RTQSPRRR	922,979	HBV	4.146	1.83	− 1.322460579	0.331900001
19	*TMVMELIRMIK	566,925	Influenza A	4.228	0.86	− 0.993923432	0.748042839
20	*TVVGRRGRSPR	N.D	HBV	4.961	1.12	− 0.506637027	1.259450078
21	*RSQSPRRR	N.D	HBV	6.236	1.07	− 1.32582118	0.185850024
22	*DLAQCFFCFK	922,790	Human	6.283	0.93	− 0.822543625	0.395600021
23	*MVWESGCTV	140,054	Human	9.834	0.3	− 1.156943761	0.593700051
24	*TVIRRRGR	923,033	HBV	10.848	0.61	− 1.025365624	0.148200035
25	*TVVRRRCRSPR	N.D	HBV	11.401	1.39	− 0.974649089	0.414450049
26	*FLLTKILTI	16,753	HBV	15.359	0.26	− 1.236918075	0.179100007
27	*TVVRRRCR	N.D	HBV	16.528	0.8	− 1.340376738	0.462499976
28	*NILNPFLPLL	N.D	HBV	16.986	0.29	− 1.178306492	0.282749981
29	*LNIDLLWSV	103,368	Human	17.661	0.29	− 1.081006694	0.273800016
30	*GLSPTVWLSA	21,137	HBV	18.362	0.35	− 1.259120664	0.248750001
31	*FIDSYICQV	137,402	Human	20	0.25	− 1.125312408	0.349050045
32	*VYALPLKML	71,976	Herpes virus	20.967	0.23	− 0.923903128	− 0.021949947
33	*LLVPFVQWFV	37,919	HBV	25.231	0.3	− 1.193666275	0.19264999
34	*AYQKRMGVQM	175,777	Influenza A	26.167	0.25	− 1.01135862	− 0.033149958
35	*LLAQFTSAL	N.D	HBV	30.833	0.25	− 1.231250251	0.213950008
36	*EYLVSFGVW	15,061	HBV	40.5	0.23	− 1.034783308	− 0.014099956

Peptides that are not stabilizing in at least two out of three stabilizing methods are marked with *. Also, a poorly predicted NetMHCpan peptide 16, which is stabilizing is marked with #. The instability cutoffs for the three stabilizing-based methods are NetMHCstab < 2 h, EZ MHC-I < 0.3 and (ELISA < 0.6 is based on non-HLA-A11:01 peptide 23 (MVWESGCTV), a known HLA-A*02:01 antigen but not non-validated peptide 20).

*EBV* Epstein–Barr virus, *DENV* Dengue virus, *N.D* denotes not determined.

Next, to enrich the experience with the EZ MHC-I assay using the HLA-A*11:01 single chain protein, a total of 180 overlapping 8- to 12-mer peptides from the epidermal growth factor receptor (EGFR) driver mutations were designed. However, three peptides, QLMPFGSLL, PFGSLLDYV and GICLTSTVQLIM are unstable and thus excluded. The remaining 177 peptides are derived from exon 19 deletion (E746-A750 at the Leu-Arg-Glu-Ala sequence, EX), exon 21 mutation (L858R, LR) and exon 20 mutations (T790M, TM and C797S, CS), which are associated with a number of cancers including lung cancer^14^ (Table 3; Supplementary Table S2). Here, the same pattern of destabilizing EZ_50kDa_ trending downward wsith increasing NetMHCpan ranks is observed (Fig. 2f; Supplementary Data 1). More importantly, with a broader peptide pool, two peptides CS1017 and CS1032 had similar measured stability as the antigenic Epstein–Barr virus peptide EBV1101 positive control but surprisingly not with NetMHCpan-4.0 and NetMHCstab-1.0 algorithms (Fig. 2f,g). Other possible exceptions include peptides EX1018 and LR1044 with very poor NetMHCpan ranks but are marginally stabilizing with an EZ_50kDa_ score of 0.2 (Table 3). Although, the EZ_50kDa_ cutoff is currently set at 0.3 (Supplementary Fig. S3), the exact minimum threshold of EZ_50kDa_ score and peptide antigenicity remains undetermined but these peptides are likely to escape thymic negative selection and be presented as pMHC-I complexes with varying density on non-professional antigen presenting cells.

**Table 3:** HLA-A*11:01 peptides comparison derived from EGFR mutations.

Index	Peptide ID	Sequence	EZ_50kDa_	EZ_50kDa_ rank out of 177	NetMHCpan-4.0 rank HLA-A*11:01 (%)	NetMHCstab-1.0 score HLA-A*11:01 (h)
–	EBV1101	IVTDFSVIK	1	–	0.0423	14.9481
1	CS1032	QLMPFGSLLDY	0.869 ± 0.174	1	3.7905	0.3434
2	LR1011	KITDFGRAK	0.859 ± 0.003	2	0.14	9.3274
3	CS1027	SLLDYVREHK	0.811 ± 0.171	3	0.4221	8.8147
4	CS1017	SLLDYVREH	0.733 ± 0.140	4	3.2	0.7919
5	CS1037	GSLLDYVREHK	0.367 ± 0.022	5	2.67	2.2755
6	EX1018	VAIKTSPKANK	0.222 ± 0.010	6	2.5	2.1099
7	LR1044	KITDFGRAKLLG	0.168 ± 0.030	7	30.7	0.5073
8	TM1050	MQLMPFGCLLDY	0.0956 ± 0.004	8	15.2	0.2965
9	LR1027	RAKLLGAEEK	0.0574 ± 0.002	9	1.8	0.8338
10	LR1009	HVKITDFGR	0.0553 ± 0.003	10	2.64	0.7457
11	CS1036	FGSLLDYVREH	0.0420 ± 0.010	11	49	0.2613
12	LR1032	KITDFGRAKLL	0.0352 ± 0.024	12	20.4	0.2729
13	EX1003	KTSPKANK	0.0341 ± 0.022	13	2.45	5.6887
14	CS1050	SLLDYVREHKDN	0.0255 ± 0.005	14	20.85	0.4105
–	EBV0201	CLGGLLTMV	0	–	46.6	0.2538
–	EX1012	AIKTSPKANK	− 0.007 ± 0.004	20	0.88	1.9707
–	LR1020	VKITDFGRAK	− 0.056 ± 0.059	77	1.6	0.5672
–	LR1003	ITDFGRAK	− 0.052 ± 0.001	63	3.45	1.4645
–	LR1030	HVKITDFGRAK	− 0.004 ± 0.022	18	3.6	2.0781
–	CS1022	LMPFGSLLDY	− 0.058 ± 0.012	80	3.655	0.2472

The top 14 peptides from EGFR mutations out of 177 peptides ranked using the EZ MHC-I assay and HLA-A*11:01 single chain protein. Five poorly scored peptides with EZ_50kDa_ less than 0.1 and NetMHCpan rank less than 5% are also included for comparison. *EBV* Epstein–Barr virus, *EX* exon 19 deletion, *LR* L858R, *CS* C797S, *TM* T790M. The EZ_50kDa_ score shows that some poorly predicted NetMHCpan-4.0 peptides (> 2%) could be stabilizing epitopes. The scores from the NetMHCstab-1.0 algorithm are also shown for comparison.

Last, based on the observed good agreement between EZ MHC-I assay and existing methods highlighted in this work using the well studied HLA-A*1101 allele, understudied HLA alleles may finally be explored. Presently, understudied HLA alleles cannot be accurately predicted due to insufficient public data to develop suitable in silico algorithm and unfavorable low-throughput methods. Here, an understudied but frequent Asian allele HLA-A*02:07 was evaluated using the rapid EZ MHC-I assay. The top eight predicted HLA-A*02:07 peptides from the same library of 177 EGFR peptides were selected using NetMHCpan-4.0 algorithm. Also, EBV1101 and Hepatitis B peptide HepB0207 were used as control peptides; EBV1101 is a predicted non-binder and HepB0207 is a known binder^15^. Using the HLA-A*02:07 single chain protein, out of the eight EGFR-derived peptides, only CS1044 and EBV0201 peptides were found stabilizing (Fig. 2h). CS1044 has a poor NetMHCpan rank of 2.238% and EBV0201 is an original HLA-A*02:01 binder and thus may be a shared cytotoxic T lymphocyte epitope with HLA-A*02:07. Also EBV0201 (CLGGLLTMV) and CS1044 (QLMPFGSLLDYV) are unlike classical HLA-A*02:07 peptides which tend to have D/P in position 3^15^. These suggest that more remains to be learned from the highly polymorphic HLA-alleles. Hence understudied but important HLA alleles remain inaccurate in silico but may be evaluated with the EZ MHC-I assay without sacrificing precious cells.

## Discussion

The EZ MHC-I assay framework largely depends on targeted proteolysis of destabilized protein. Proteolysis can occur non-enzymatically and enzymatically. However, non-enzymatic thermal proteolysis is inefficient with prominent α and β2m chains of unchained MHC-I protein still present even at a higher temperature 37 °C. Although most single chain pMHC-I remain thermal stable, some single chain pMHC-I proteins such as the HLA-A*02:07 protein described in this work can be thermally proteolyzed but still usable, as rescued pMHC-I proteins are thermostable at 37 °C^10^. Based on an enzyme-exchangeable single chain pMHC-I molecular design, a cleavable bulging peptide allows peptide exchange. More importantly, enterokinase was repurposed to target accessible sites such as intrinsically disordered regions in a destabilized α chain and also free peptides^16^. In this work, the non-specificity of enterokinase is shown at 37 °C towards destabilized proteins but not stable and folded pMHC-I single chain protein.

Enterokinase is naturally secreted by the Brunner’s glands for digestion in the duodenum and plays no known biological role in antigen presentation by the MHC-I proteins. However, in this technology, the non-specificity of enterokinase was successfully harnessed to cleave residues in the destabilized α chain not bearing the canonical DDDD|K motif but exposed by a leaving β2m chain attached. The enchained β2m is needed to further destabilize the α chain, as most of the empty α chain remains undigested for unchained pMHC-I protein even at 37 °C. The broad pH range of enterokinase activity between 6.0 and 8.5 also favors screening at pH 6.2 close to the luminal pH and peptide exchange above room temperature^17,18^. Moreover in this work, obvious protein fragments due to non-rescued single chain MHC-I protein with enterokinase is seen at 37 °C. Taken together, the EZ MHC-I assay for the highly polymorphic single chain pMHC-I proteins may be performed under mildly acidic luminal conditions similar to the endosomal compartments^17^.

The pMHC-I protein stability is well regarded as a better predictor than affinity as immunogenic peptides which bind more stably, also better define CD8^+^ T cells^7^. Also, Parker et al. has shown that the loss of ^125^I-labeled β2m strongly reflects peptide dissociation in the α chain^9^. However, the lack of simple stability-based assays has continued worldwide usage of inaccurate affinity-based methods. Here, the use of a single chain construct propels whole (empty) MHC-I protein instability and induced protein fragments by enterokinase. This rapid technology has shown that NetMHCpan-4.0 predicted strong peptide binders (< 0.5%) and NetMHCstab-1.0 predicted stable peptides (> 2 h) generally also mean stable pMHC-I proteins. However, weaker and non-canonical peptides remain challenging for current in silico algorithms. In particular, lengthy peptides i.e. 15- and 16-mer, remain unpredicted and have been isolated in HLA class I molecules with potential roles in T cell immunity^19–21^. Although lengthy peptides or even mix-match combination remain unexplored here, the more versatile EZ MHC-I assay is feasible for studying lengthy peptides, post-translated peptides and dual peptide occupancy.

Here, the EZ MHC-I assay for HLA-A*11:01 has identified a set of 14 stabilizing peptides with varying NetMHCpan ranks derived from key EGFR mutations associated with non-small cell lung cancer (NSCLC). Approximately 80% of lung cancers is associated with NSCLC driven by molecular EGFR mutations and ALK receptor tyrosine kinase translocations^22,23^. For somatic EGFR mutations, classical exon 19 deletion and L858R represent the majority of EGFR mutations in NSCLC, and are positive prognostics towards specific EGFR tyrosine kinase inhibitors (TKIs), gefitinib and erlotinib^24,25^. However, mutants such as T790M and C797S are commonly associated with rapidly acquired resistance during TKI treatment and thus a need for new strategies that specifically target such driver mutations^26^. Hence an alternative strategy to the classical ATP-site of the kinase is T cell biology. Unlike MHC-II, which is not expressed by most epithelial cells, MHC-I is expressed on most nucleated cells. More importantly, EGFR inhibitors such as erlotinib, cetuximab, and nimotuzumab can enrich peptide-MHC density on the skin (and likely other organs) and recruit T cell-driven processes^27^. Thus identifying relevant antitumor CD8^+^ T cells is a mean to increase clinical efficacy.

In summary, EZ MHC-I assay highlights the repurposing of intestinal enterokinase towards destabilized protein not bearing the standard Asp-Asp-Asp-Asp-Lys motif to significantly improve MHC-I peptide selection for pMHC-I multimer technology. Conceptualizing enterokinase in a broader context will reshape other protein instability-based assays. Here, in silico predictions are still limited to well-studied HLA-alleles, canonical peptides and affinity binding based data. The results from stability-based EZ MHC-I assay could help advance the development of in silico tools and discover more peptide epitopes across health and diseases.

## Methods

### Production of single chain pMHC-I protein

A melittin-leader modified MultiBac acceptor pACEBac1 vector is used to make the secreted single chain pMHC-I protein. In the single chain trimer design, the unique BamHI and XbaI restriction sites introduce a peptide center within the peptide-β2m-α single chain module separated by two GS-rich spacers, and a peptide bearing DDDD|K peptide sequence to aid peptide dissociation and exchange. Following standard bacmid preparation and infection of insect cells, different single chain pMHC-I proteins are overexpressed as secreted proteins in Spodoptera *frugiperda* (Sf9) or High Five cells. The secreted single chain pMHC-I proteins bearing a 6x-His tag are purified by tandem Ni–NTA and size-exclusion column purification in 20 mM Tris pH 8 and 150 mM NaCl buffer.

### EZ-peptide screening

Purified single chain pMHC-I proteins (~ 50 kDa) are treated with enterokinase (NEB P8070) in 20 mM sodium cacodylate pH 6.2 and 150 mM NaCl buffer or 20 mM Tris pH 8.0 and 150 mM NaCl buffer at 37 °C for evaluating peptide exchange and induced whole protein fragmentation with Laemmli SDS-PAGE without boil.

### Peptide synthesis

Lypholized peptides are synthesized by Mimotopes with at least 90% purity and stored as 10 mM dimethyl sulfoxide stocks for long-term storage at − 80 °C.

### ELISA assay with photolabile unchained pMHC-1 protein and UV irradiation

The ELISA is performed using a photolabile pMHC-I protein mixture containing both biotinylated and non-biotinylated proteins. This is to minimize false positive due to aggregated empty MHC-I protein, whereby an excess of non-biotinylated proteins at 97.5:2.5 molar ratio to biotinylated protein will minimize the formation of non-specific biotinylated protein aggregates. Non-biotinylated photolabile pMHC-I proteins refolded from the E. coli expression system were also purified with Dynabeads-streptavidin to remove endogenous biotinylated pMHC-I protein. For the ELISA setup, 400 ng of anti-human β2m (BioLegend, cat no. 316302) in ELISA coating buffer (BioLegend, cat no. 421701) was coated onto Nunc Maxisorp ELISA plates (BioLegend, cat no. 423501) at 4 °C overnight. The next day, unbound anti-human β2m was washed out with 1xELISA wash buffer (BioLegend, cat no. 421601) diluted in water and blocked with 1 × ELISA diluent B (BioLegend, cat no. 421205) diluted in phosphate buffer saline (PBS) for 1 h at 25 °C. The blocking buffer was washed out with 1xELISA wash buffer and the ELISA plate tapped dry. Next, in a 96 well plate, a reactant mixture of 62.5 nM of photolabile pMHC-I protein mixture with or without 5 μM of peptide in PBS was added and irradiated on ice with UV for 2 × 5 min using the UV crosslinker chamber at 365 nm (UVP, cat no. CL-1000L). The reactant mixture was transferred to the anti-human β2m-coated ELISA and left to incubate for 2 h at room temperature. After incubation, the unbound reactant was washed out with 1xELISA wash buffer and blocked with 1 × ELISA diluent B for 1 h at room temperature. The blocking buffer was washed out with 1xELISA wash buffer and the ELISA plate was tapped dry. For detection of bound biotinylated pMHC-I protein, streptavidin–horseradish peroxidase was added and allowed to incubate for 30 min at room temperature before flicking off, washing with 1xELISA wash buffer and the ELISA plate tapped dry. Finally, ultra 3,3′5,5′-tetramethylbenzidine was added for 10 min at room temperature before quenching with equal volume of 2 M sulfuric acid. The absorbance at 450 nm was measured with a plate reference at 570 nm using the microplate reader and corrected using Eq. (1):

1 $$\Delta {\text{Abs}}_{{{45}0{\text{nm}}}} = {\text{ Abs}}_{{{45}0{\text{nm}},{\text{ uncorrected}}}} - {\text{ Abs}}_{{{57}0{\text{nm}}}}$$

### EZ MHC-I assay with single chain pMHC-I protein and enterokinase

The EZ MHC-I assay is performed in an eppendorf tube containing 3 U of enterokinase (NEB P8070) for every 1 μg of single chain pMHC-I protein with or without a peptide diluted in 20 mM sodium cacodylate pH 6.2 and 150 mM NaCl buffer and left at 37 °C for 14 h (overnight). To stop peptide exchange and proteolysis, the eppendorf tube is spun at 4 °C at 14,000×*g* for 3 min, also to settle any condensate prior to Laemmli SDS-PAGE without boil.

### Calculation for EZ MHC-I assay

The EZ MHC-I assay for a peptide library is always checked with same peptide controls performed at 37 °C to assess quality of proteins and protein gel (Supplementary Data 1). For HLA-A*11:01, the positive peptide control (IVTDFSVIK) ensures rescue upon digestion. The negative peptide control (CLGGLLTMV) represents any insignificance upon digestion when also compared to one without a peptide. For HLA-A*02:07, the control peptides are FLPSDYFPSV (positive) and IVTDFSVIK (negative). A digested control and an undigested control are used for normalization. Samples are loaded without boil onto TGX stain-free precast gel (Bio-Rad) for Laemmli SDS-PAGE and imaged with the ChemiDoc MP imaging system (Bio-Rad). The protein band volume (*v*) is extracted using the Image Lab software version 6.1 (Bio-Rad, https://www.bio-rad.com/en-sg/product/image-lab-software?ID=KRE6P5E8Z):

2 $${\text{Normalized Positive Peptide Control}}_{{{5}0{\text{kDa}}}} = \frac{{V_{dig}^{ + peptide} - V_{dig} }}{{V_{undig} - V_{dig} }}$$

3 $${\text{Normalized Negative Peptide Control}}_{{{5}0{\text{kDa}}}} = \frac{{V_{dig}^{ - peptide} - V_{dig} }}{{V_{undig} - V_{dig} }}$$

$${\text{Undigested}}_{{{5}0{\text{kDa}},{\text{ stable}}}} = \frac{{V_{undig} - V_{dig} }}{{V_{undig} - V_{dig} }} = 1 \quad \left( {{\text{default}}} \right)$$

$${\text{Digested}}_{{{5}0{\text{kDa}},{\text{ unstable}}}} = \frac{{V_{dig} - V_{dig} }}{{V_{undig} - V_{dig} }} = 0\quad \left( {{\text{default}}} \right)$$

The positive and negative peptide controls also score the EZ pMHC-I stability (EZ_50kDa_) of each rescue peptide ranging from above 1 (more stable than control), 1 (equally stable) to below 0 (very unstable), and is determined by:

4 $${\text{EZ}}_{{{5}0{\text{kDa}}}} = \frac{{V_{dig}^{unknown} - V_{dig}^{ - peptide} }}{{V_{dig}^{ + peptide} - V_{dig}^{ - peptide} }}$$

### In silico peptide prediction

Peptides were predicted using either the NetMHCpan-4.0 algorithm at https://services.healthtech.dtu.dk/ or the NetMHCstab-1.0 algorithm at https://www.cbs.dtu.dk/services/NetMHCstab/^13,28^.

## Supplementary information

Supplementary file1

Supplementary file2

## Data Availability

All data collected for the EZ MHC-I assay are included in the paper and/ or the Supplementary Material. Additional data related to the paper may be requested from J.L.

## Code availability

NetMHCpan-4.0 algorithm is available from https://services.healthtech.dtu.dk/. NetMHCstab-1.0 algorithm is available from https://www.cbs.dtu.dk/services/NetMHCstab/. Calculation used for scoring EZ MHC-I stability (EZ_50kDa_) is described in this paper and may also be found in Supplementary Data 1.
