# Supplementary material for: Destabilizing single chain major histocompatibility complex class I protein for repurposed enterokinase proteolysis
Source: Sci Rep. 2020 Sep 10;10:14897. doi: 10.1038/s41598-020-71785-2 (PMC7483518; doi:10.1038/s41598-020-71785-2)
Supplement: Supplementary file 2 — Supplementary file2 [file 41598_2020_71785_MOESM2_ESM.docx]

**Supplementary Information**

**Destabilizing Single Chain Major Histocompatibility Complex Class I Protein for Repurposed Enterokinase Proteolysis**

Jackwee Lim*

Singapore Immunology Network, A*STAR, 8a Biomedical Grove, Singapore 138648

*Correspondence to: lim_jack_wee@immunol.a-star.edu.sg

**This PDF file includes:**

Supplementary Fig. 1

Supplementary Fig. 2

Supplementary Fig. 3

Supplementary Tables 1 and 2

**Other Supplementary Materials for this manuscript include the following:**

Supplementary Data S1 [Data_S1.xls]

**Supplementary Fig. 1.**

**Supplementary Figure 1.** **Selected EGFR peptide library** **evaluation of unchained and single chain HLA-A*11:01 MHC-I protein constructs at pH 6.2 and 37 °C. a**, Photo-labile unchained MHC-I protein was exposed to long-wavelength UV for 10 min on ice to digest the photo-labile peptide with or without enterokinase (EK), and incubated for 14 h. UV treatment is performed with the CL-1000 Ultraviolet Crosslinker (Analytic Jena AG, Germany) for 10 min on ice. A plot of raw data of the photo-labile unchained protein treated with peptides and enterokinase for 16 h and showed non-interpretable assay based on protein band volume intensities of controls (white bars) and no rescue across the peptide candidates. **b**, A plot of raw data of the single chain protein treated with peptides and enterokinase for 14 h showing rescued, non-rescued pMHC-I proteins and about 90 % digested fragments at 37 °C. The protein gel lanes in (**a**) and (**b**) are arranged in the order: MW ladder, EBV1101, CS1027, CS1032. CS1017, CS1037, LR1011, EX1018, TM1050, CS1050, CS1048, EBV0201, undigested kept at 25 °C, undigested kept at 37 °C and digested kept at 37 °C. The positive EBV1101 and negative EBV0201 peptide controls are marked with (*) in the protein gels.

**Supplementary Fig. 2.**

**Supplementary Figure 2.** **Single chain MHC-I protein gels of inserts shown in figure 2. a,** Full gel of insert shown in 2d showing HLA-A*11:01 single chain protein bands corresponding to EZ MHC-I assay treatment with peptides in the order of 1. Protein ladder 2. IVTDFSVIK 3. SVQPTFSVDQR 4. AYQKRMGVQM 5. LYASPQLEGF 6. LVTFLLLCGR 7. FTNDSIISH 8. CLGGLLTMV 9. Repeat IVTDFSVIK 10. AYQKRMGVQM 11. LVTFLLLCGR 12. CLGGLLTMV 13. Undigested protein kept at 25 °C 14. Undigested protein kept at 37 °C 15. Digested protein without peptide kept at 37 °C. **b,** Full gel of insert shown in 2g showing thermolabile HLA-A*02:07 single chain protein bands corresponding to EZ MHC-I assay treatment with peptides in the order of 1. Protein ladder 2. HepB0207 (FLPSDYFPSV) 3. CS1020 (TQLMPFGSLL) 4. CS1030 (ITQLMPFGSLL) 5. TM1011 (STVQLIMQL) 6. LR1012 (ITDFGRAKL) 7. EBV0201 (CLGGLLTMV) 8. TM1027 (MQLMPFGCLL) 9. CS1044 (QLMPFGSLLDYV) 10. CS1041 (LITQLMPFGSLL) 11. CS1002 (QLMPFGSL) 12. EBV1101 (IVTDFSVIK) 13. Undigested protein kept at 25 °C 14. Undigested thermolabile protein kept at 37 °C 15. Digested thermolabile protein kept at 37 °C.

**Supplementary Fig. 3**

**Supplementary Figure 3. The EZ_50kDa_ cutoff at 0.3.** From the Immune Epitope Database (IEDB, https://www.iedb.org/), retrieved peptides listed in Table 1 were ranked based on predictive algorithms. (a) A cutoff of EZ_50kDa_ cutoff at 0.3 included all 27 peptides with NeMHCspan rank < 1 % at the preferred pH 6.2. (b) A cutoff of EZ_50kDa_ cutoff at 0.3 included all 16 peptides with NeMHCstab rank greater than 2 h at the preferred pH 6.2.

**Supplementary Table 1.**

| **Index** | **Sequence** | **EZ_50kDa_**  **rank** | **NetMHCpan-4.0**  **rank (%)** | **ForestMHC**  **rank** |
| --- | --- | --- | --- | --- |
| 13 | RQLANAIFK | 1.303 | 0.17 | 0.797 |
| 24 | MSYTMCSGK | 1.198 | 0.62 | 0.841 |
| 1 | ATIGTAMYK | 1.032 | 0.003 | 0.984 |
| 10 | AINSEMFLR | 0.901 | 0.134 | 0.787 |
| 12 | TSGSPIIDK | 0.795 | 0.169 | 0.959 |
| 26 | FTNDSIISH | 0.696 | 0.798 | 0.416 |

**Supplementary Table 1. HLA-A*11:01 9-mer peptide ranks in EZ MHC-I assay, NetMCHpan-4.0 and ForestMHC binding algorithms**. No prediction is available with ForestMHC algorithm for some 9-mers; ATYGWNLVK, STYGWNIVK, IVTDFSVIK, ATVQGQNLK, KTFVDLMRR, KSGAIKVLK, MVSRLLLNR, GPISGHVLK and CLGGLLTMV. Data for EZ MHC-I assay with HLA-A*11:01 single chain protein was collected at pH 6.2 at 37 °C.

**Supplementary Table 2.**

| **EGFR mutations** | **Original Template** | **Mutated Template** |
| --- | --- | --- |
| EX19-Deletion | KIPVAIK**ELREA**TSPKANKEILDE | KIPVAIKTSPKANKEILDE |
| L858R | QHVKITDFG**L**AKLLGAEEKEY | QHVKITDFG**R**AKLLGAEEKEY |
| T790M | GICLTSTVQLI**T**QLMPFGCLLDY | GICLTSTVQLI**M**QLMPFGCLLDY |
| C797S | VQLITQLMPFG**C**LLDYVREHKDN | VQLITQLMPFG**S**LLDYVREHKDN |

**Supplementary Table 2. Sequence templates of EGFR mutants used to derive 177 overlapping peptides in Data S1.**

**Supplementary Data S1.**

**Supplementary Data S1. Raw data of 177 EGFR peptides used in this work.**
